# Sensor-Based Daily Physical Activity: Towards Prediction of the Level of Concern about Falling in Peripheral Neuropathy

**DOI:** 10.3390/s20020505

**Published:** 2020-01-16

**Authors:** Gu Eon Kang, Bijan Najafi

**Affiliations:** Interdisciplinary Consortium on Advanced Motion Performance (iCAMP), Michael E. DeBakey Department of Surgery, Baylor College of Medicine, Houston, TX 77030, USA; gueon.kang@bcm.edu

**Keywords:** peripheral neuropathy, physical activity, concern about falling, eHealth, wearable sensor, walking behavior, standing behavior, sedentary behavior, walking episodes, step counts

## Abstract

Concern about falling is prevalent and increases the risk of falling in people with peripheral neuropathy (PN). However, the assessment of concern about falling relies on self-report surveys, and thus continuous monitoring has not been possible. We investigated the influence of concern about falling on sensor-based daily physical activity among people with PN. Forty-nine people with PN and various levels of concern about falling participated in this study. Physical activity outcomes were measured over a period of 48 hours using a validated chest-worn sensor. The level of concern about falling was assessed using the falls efficacy scale-international (FES-I). The low concern group spent approximately 80 min more in walking and approximately 100 min less in sitting/lying compared to the high concern group. In addition, the low concern group had approximately 50% more walking bouts and step counts compared to the high concern group. Across all participants, the duration of walking bouts and total step counts was significantly correlated with FES-I scores. The duration of walking bouts and total step counts may serve as eHealth targets and strategies for fall risk assessment among people with PN.

## 1. Introduction

In the United States, an estimated 30 million people suffer from some form of peripheral neuropathy (PN), most commonly diabetic PN and chemotherapy-induced PN [1]. PN is a major risk factor for falling due to damages in the sensory nerve in the extremities [2]. For example, the likelihood of falling is 23 times more for people with diabetic PN compared to those without [3]. In addition, people with chemotherapy-induced PN are three times more likely to fall compared to those without [4]. The greater likelihood of falling associated with PN may contribute to the development of a concern about falling [5], which subsequently leads to reduced physical activity in affected people in order to avoid fall-related injuries [6]. This restricted physical activity may worsen their physical health and consequently increase their risk of falling in the long term [7,8].

Currently, an assessment of concern about falling is primarily based on itemized self-report surveys such as the falls efficacy scale-international (FES-I) [9]. Although its validity has been well-established previously [9,10], relying on self-report surveys has some critical limitations. First, it is difficult to monitor concern about falling continuously and in real time. In fact, it was argued in previous studies that less frequent assessments of the risk of falling may lead to inaccurate classification of this risk [11,12]. Second, it is unclear how the level of concern about falling actually affects physical activity. Because physical activity is often measured using self-report surveys that primarily rely on subjects’ recall of memory, it is questionable whether the restricted physical activity is a perceived or a real symptom. In a recent review article, it has been argued that objective measures of physical activity provide clinically more meaningful information compared to subjective measurement [13]. Therefore, there is a critical need to develop new biomarkers of the severity and progression of concern about falling along with low-cost, widely accessible tools for their measurement.

These limitations may be addressed by using smart wearable sensors for eHealth applications, as they provide objective information that is potentially relevant to concern about falling. For example, commercial activity trackers provide objective, quantitative, and continuous information about physical activity measures such as total daily activity and total step counts, which is likely to decrease with the concern about falling [14]. These sensors have been used in previous studies, and successfully demonstrated characteristics of physical activity in people with diabetic PN [15,16,17,18,19]. Although, concern about falling is prevalent in PN, to date, no study has reported how the level of concern about falling among people with PN is associated with objective physical activity outcomes. 

In terms of physical activity outcomes, most of the studies that investigated daily physical activity and the risk of falling in vulnerable populations focused on broad measures such as total daily activity or step counts [20,21]; however, simply reporting such outcomes may not provide enough information regarding concern about falling. It was suggested in a recent study that investigating more detailed patterns or variabilities of physical activity such as time that subjects spent walking or sedentary behavior may provide more clinically meaningful information than simply reporting total daily activity or step counts [22].

The aim of this study was to investigate the influence of concern about falling on objectively measured daily physical activity among people with PN. Daily physical activity was measured over a 48-hour period continuously using a wearable inertial sensor. Physical activity outcomes were the duration of walking bout, and standing and sedentary postures, total number of walking bouts, and total step counts over the 48-hour period. Based on previous findings about concern about falling and subjectively measured physical activity level in people with PN [14], we hypothesized that sensor-based physical activity outcomes would identify the level of concern about falling among people with PN.

## 2. Materials and Methods

### 2.1. Participants

Forty-nine people participated in this study. Inclusion criteria were a diagnosis of PN confirmed by their physicians, and daily symptoms of PN such as numbness and burning pain in their feet. We excluded people who had a history of a neurological disorder such as Parkinson’s disease and dementia or had an active foot ulcer or foot infection, because they might have affected the levels of physical activity. The ages of the participants ranged between 56 and 85 years, and causes of PN were diabetes mellitus (N = 23) and chemotherapy (N = 26). All participants provided written informed consent that was approved by the Institutional Review Board of the Baylor College of Medicine.

### 2.2. Clinical Measures

We used the FES-I in order to evaluate participants’ concern about falling [9]. The FES-I is a 16-item self-administered questionnaire that assesses the level of concern about falling during day-to-day activities such as house chores, stair climbing, and walking. Scores on each item range between 1 (“not at all concerned”) and 4 (“very concerned”), and total scores between 16 and 19, between 20 and 27, and between 28 and 64 on the FES-I indicate low, moderate, and high concern about falling, respectively [10].

In order to assess the severity of PN, we measured vibration perception threshold (VPT) in the plantar surface of the foot (the first and fifth metatarsal heads, and the heel) using a standard biothesiometer (Bio-Medical Instrument, Newbury, Ohio, United States) as performed in previous studies [23,24,25,26,27]. Briefly, the probe of the biothesiometer was placed on the plantar surface of the foot, and electrical vibration was gradually increased from 0 volts until participants felt the electrical vibration. The vibration continued to increase slightly from the magnitude that participants felt, and then was decreased until participants no longer felt the vibration. We repeated this procedure until the two magnitudes were close (<2 volts). Additionally, participants self-disclosed their fall history in the past 12 months.

### 2.3. Sensor-Based Measures of Physical Activity

**Figure 1:**
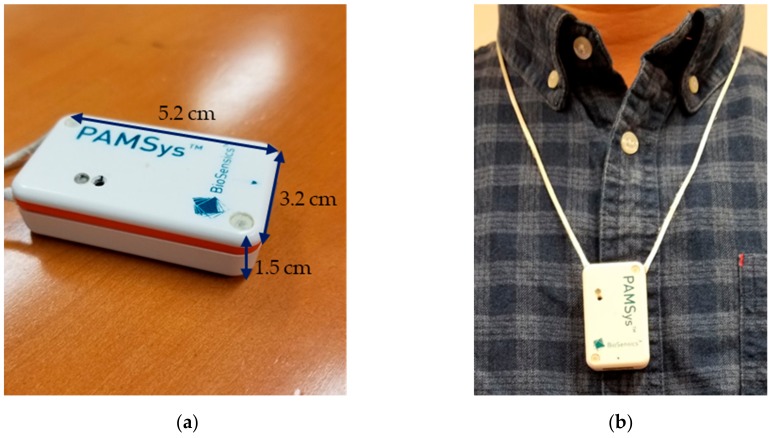
The wearable sensor that was used for measuring daily physical activity: (**a**) it is small (5.2 cm length × 3.2 cm width × 1.5 cm height) and light-weight (20 grams) and (**b**) it was worn on the chest as a pendant necklace.

We measured daily physical activity over a 48-hour period using a commercial wearable sensor (PAMSys^TM^, BioSensics LLC, Newton, MA, USA) that was worn on the chest (Figure 1). The sensor was worn during the total 48-hour period except for water activity (e.g., shower) and sleep. We asked participants to provide a daily log of times and durations for the water activities and sleep. In addition, all participants were instructed not to touch the sensor except for when taking off and wearing in order to avoid unnecessary oscillations. The sensor consisted of a tri-axial accelerometer (±2 g) and collected linear acceleration of the trunk with a sampling rate of 50 Hz. The collected data were filtered using a wavelet filter bank with a cut-off frequency of 12.5 Hz [28]. Commercial software (PAMWare^TM^, BioSensics LLC, Newton, MA, USA) was used to calculate duration of walking bouts, standing and sedentary postures, the number of walking bouts, and total step counts over the 48-hour period. The algorithm and validity of the sensor for physical activity measurements has been described elsewhere [29,30,31,32,33,34]. Briefly, durations for standing, sitting, and lying cumulative postures were estimated based on a simple biomechanical model of the chest and determination of the type of transitions between two postures (e.g., from sitting to standing) as well as a series of biomechanical rules described elsewhere (e.g., prolonged leaning backward of the chest is unlikely during standing, and walking is impossible during sitting) [29,30,31]. Walking bouts were calculated from a peak detection algorithm from vertical acceleration, and a walking bout was defined as consisting of a minimum of three consecutive steps [31]. Finally, we defined sedentary postures as a combination of lying and sitting postures.

### 2.4. Statistical Analysis

The primary purpose of this study was to investigate the influence of the level of concern about falling on objectively measured daily physical activity. Thus, we classified the participants into three groups based on their FES-I scores: a group with low concern about falling (low concern group), another group with moderate concern about falling (moderate concern group), and another group with high concern about falling (high concern group). For demographic and clinical characteristics, we compared mean values among the three groups using one-way analysis of variance (ANOVA) for body-mass index and VPT (i.e., parametric variables) using Kruskal-Wallis tests for age, FES-I, and number of falls in the past 12 months (i.e., nonparametric variables), and using Chi-square tests for number of women and number of people who had a fall incidence in the past 12 months (i.e., categorical variables).

For physical activity outcomes, we first performed cross-sectional comparisons using one-way ANOVA (Tukey corrections for post-hoc pairwise comparisons) among the three groups (after checking normality using Shapiro-Wilk tests). Then, across all participants, we examined correlations between FES-I scores and physical activity outcomes using Spearman correlations (R_s_). For all statistical analyses, a *p*-value < 0.05 was considered statistical significance.

## 3. Results

### 3.1. Demographic and Clinical Characteristics

We summarized demographic and clinical characteristics for each group and across all participants in Table 1. Among the forty-nine participants, based on FES-I, fifteen, twelve, and twenty-two participants had low, moderate, and high concern about falling, respectively. There were no significant differences in age, the number of women, and VPT among the three groups (all *p* > 0.05). As expected, the low concern group had 27.5% and 59.8% lower FES-I compared to the moderate and high concern groups, respectively (*p* < 0.001). The number of fall incidences during the past 12 months was 6 and 11 times more for the moderate and high concern groups, respectively, than for the low concern group (*p* = 0.009). The number of people who fell at least once in the past 12 months was higher in the high concern group compared to the other two groups. BMI was lower for the moderate concern group than for the low and high concern groups (*p* = 0.039).

### 3.2. Physical Activity Characteristics

**Figure 2:**
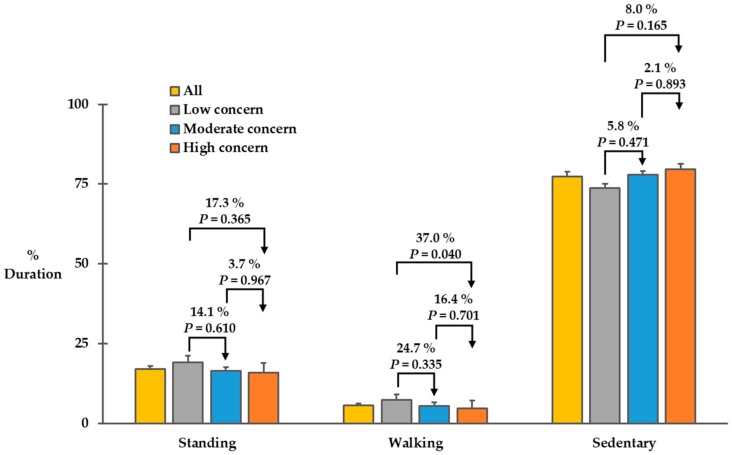
Percent durations of standing posture, walking bouts, and sedentary posture over the 48-hour period for each group. Error bars represent standard errors. *p*-values for all pairwise comparisons were adjusted with Tukey corrections.

We illustrated durations of standing posture, walking bouts, and sedentary posture in Figure 2. We found a marginal difference in the duration of walking bouts among the three groups (*F*(2,46) = 3.178, *p* = 0.051). The low concern group spent 7.3 ± 2.7% of the 48-hour period (approximately 210 min) walking, but the moderate and high concern groups spent 5.5 ± 1.0% (approximately 158 min) and 4.6 ± 0.5% (approximately 132 min) of the 48 hours-period walking, respectively. Across all participants, mean percent duration of walking bouts over the 48-hour period was 5.7 ± 0.5 % (approximately 164 min). The durations of standing posture and sedentary posture tended to be different among the three groups, but the differences did not reach statistical difference (*F*(2,46) = 0.979, *p* = 0.383 for the duration of standing posture; *F*(2,46) = 1.748, *p* = 0.186) for the duration of sedentary posture. The low, moderate, and high concern groups spent 19.0 ± 2.2% (approximately 547 min), 16.4 ± 1.7% (approximately 472 min), and 15.8 ± 1.3% (approximately 455 min) of the 48-hour period in standing. In addition, the low, moderate, and high concern groups spent 73.7 ± 3.1% (approximately 2122 min), 78.0 ± 2.5% (approximately 2246 min), and 79.6 ± 1.7% (approximately 2292 min) of the 48-hour period in sitting/lying.

We summarized characteristics of walking bouts in Table 2. Across all participants, total walking bouts and total step counts over the 48-hour period were 440 ± 36 and 9377 ± 826, respectively. Overall, the number of walking bouts and total step counts over the 48-hour period tended to be different among the three groups (*F*(2,46) = 2.231 and *p* = 0.119 for total walking bouts; *F*(2,46) = 2.195 and *p* = 0.123 for total step counts). For pairwise comparisons, the number of total walking bouts and total step counts over the 48-hour period tended to be 31.4% and 31.7% higher for the low concern group than for the moderate concern group, respectively (*p* = 0.360 and 0.401, respectively); 45.0% and 49.6% higher for the low concern group than for the high concern group, respectively (*p* = 0.105 and 0.106, respectively); and 10.3% and 13.6% higher for the moderate concern group than for the high concern group, respectively (*p =* 0.894 and 0.856, respectively); however, the differences did not reach statistical differences.

### 3.3. Correlations between FES-I Scores and Physical Activity Outcomes

**Figure 3:**
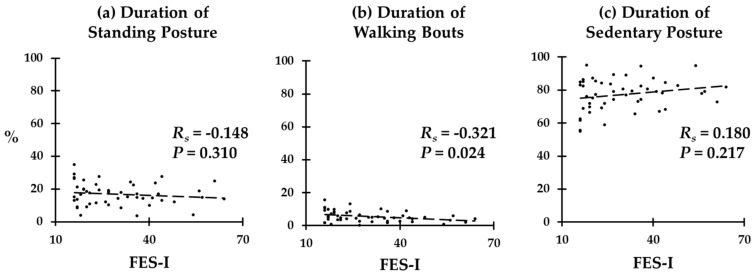
Correlations between FES-I scores, and durations of (**a**) standing posture, (**b**) walking bouts, and (**c**) sedentary posture.

We illustrated correlations between FES-I scores and durations of standing, walking, and sedentary behavior in Figure 3. We found a significant negative correlation between FES-I scores and the duration of walking behavior across all participants (R_s_ = −0.321; *p =* 0.024). The duration of standing behavior was also negatively correlated with FES-I scores (R_s_ = −0.148), and the duration of sedentary behavior was positively correlated with FES-I scores (R_s_ = 0.217); however, the correlations were not statistically significant (*p =* 0.310 and 0.217, respectively).

**Figure 4:**
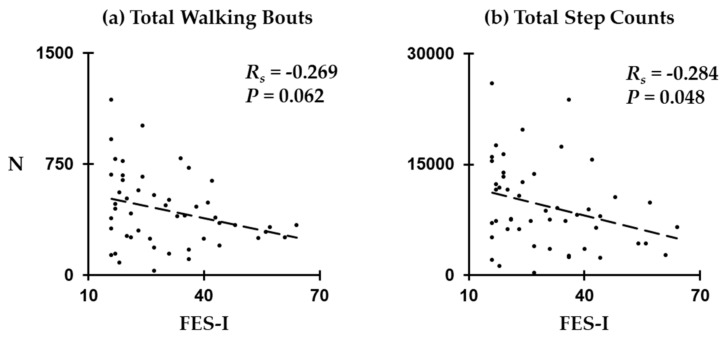
Correlations between FES-I scores and characteristics of walking behavior: (**a**) total walking bouts and (**b**) total step counts.

We illustrated correlations between FES-I scores and characteristics of walking bouts in Figure 4. We found a significant negative correlation between FES-I scores and total step counts across all participants (R_s_ = −0.284; *p =* 0.048). The number of walking bouts tended to be negatively correlated with FES-I scores (R_s_ = −0.269); however, the correlation was not statistically significant (*p =* 0.062).

## 4. Discussion

In this study, we investigated the influence of concern about falling on daily physical activity among people with PN by examining durations of standing posture, walking bouts, and sedentary posture, total number of walking bouts, and total step counts. Physical activity outcomes were measured objectively over a 48-hour period using a wearable inertial sensor. We compared the outcomes cross-sectionally among groups with low, moderate, and high concern about falling, and examined if an outcome is a predictor for the level of concern about falling. We found that all the physical activity outcomes tended to differentiate between the three groups. Particularly, there was a significant difference in the duration of walking bouts between the low concern and high concern groups. More importantly, across all participants, all the physical activity outcomes tended to be correlated with FES-I scores. Among them, we found that the duration of walking bouts and total step counts had significant correlations with the level of concern about falling. To our knowledge, this is the first to report the association between the level of concern about falling and physical activity outcomes among people with PN.

This study focused on people suffering from PN instead of general population. With global epidemic of noncommunicable disease such as diabetes mellitus and cancer, we believe that findings in our study have scientific merits. Diabetes mellitus, cancer, and other noncommunicable diseases of decay are now leading causes of global mortality in both developed and developing countries, and are consuming most of the health care resources. Timely intervention is the key to improvement, and we believe that our results will contribute to this improvement by suggesting a potential of an eHealth application (i.e., outcomes from a smart wearable sensor) for continuously monitoring the level of concern about falling and eventually the risk of falling in people who have damages in sensory nerve [35]. While mounting evidence suggests that knowledge of intra-subject performance variation can significantly augment clinical judgement and care, the majority of available neuropsychological assessments for concern about falling are ill-suited to repeat testing within relatively short periods of time due to the effects of practice, mood, fatigue, and other influences. Therefore, remote monitoring of physical biomarkers of concern about falling could help to overcome shortcomings of conventional neuropsychological assessments, and facilitate the evaluation of their severity and progression over time, along with low-cost, widely accessible tools for their measurement. For instance, the findings from this study could be embedded in eHealth technologies like wearables and mobile health applications, which would allow one to track everyday physical activities and subtle changes regarding concerns about falling over time. This in turn could assist the clinical care team to manage concerns about falling in a timely manner; to identify their reasons (e.g., plantar numbness caused by PN, poor balance because of side effects from medications, and cognitive decline) [36,37,38]; and, eventually, to reduce their consequences such as reduction in physical activities, frailty, and falls [39,40].

Our findings were in line with previous reports regarding objective physical activity and concern about falling in general population. For example, it was reported that self-reported concern about falling was significantly related to objectively measured physical activity in community-dwelling older adults (≥65 years) [41]. In this study, we found significant correlations between concern about falling, and durations of walking bouts and daily step counts, and found that concern about falling may actually decrease walking activity among people with PN. Based on previous studies that reported the association between concern about falling and less physical activity (either subjective or objective) in community-dwelling older adults [41,42,43,44], the lower amount of walking activity found in this study is likely due to concern about falling in our sample. Along with the previous report, these results confirm the effect of concern about falling in daily physical activity.

Cross-sectionally, among the three groups, the low-concern group tended to be the most active, and the high-concern group tended to be the most sedentary. A previous study reported that activity pattern is more sedentary (subjective measurement) for people with high concern about falling than for people with low concern about falling [45]. Another study reported that the level of concern about falling restricted activity level (subjective measurement) [44]. It was also reported that a decrease in the level of concern about falling was associated with improved activity level (subjective measurement) [46]. Our results were in line with these findings, and confirmed that increased sedentary behavior and decreased active behavior were real symptoms associated with concern about falling.

Similar tendency was found in total walking bouts and total step counts. Although significant differences were not found, the low concern group had the highest number of walking bouts and step counts, and the high concern group had the lowest number of walking bouts and step counts. Together with the results about the durations of standing posture, walking bouts, and sedentary posture, this evidence further confirmed reduced activity due to concern about falling among people with PN.

Many previous studies that used wearable sensors for assessing the risk of falling focused on cross-sectional results between fallers and nonfallers [47,48]. The significant correlations between FES-I scores and the duration of walking bouts, and between FES-I scores and total step counts, together with previous results for differentiating people at risk of falls, provide implications for using wearable sensors for monitoring the risk of falling.

With recent advances in wearable technology, the potential of sensor-based fall risk monitoring has been continuously discussed, and, so far, wearable technology seems to be the only possibility to objectively monitor a person’s natural behavior without limitations of time and space [49,50,51]. Our results support the argument of the potentiality of sensor-based fall risk assessment, and provide strong evidence of wearable sensors as an optimal candidate for eHealth for managing people at risk of falling.

There are some limitations in this study. One limitation is a small sample size with relatively wide range of age. A larger study with more strict age range (e.g., older adults ≥ 65 years old) is recommended to confirm the findings from this study. Another limitation is inclusion of PN due to two different causes: diabetic PN and chemotherapy-induced PN. Although their sensory function as measured using VPT was similar, it is recommended that additional studies that focus on diabetic PN or chemotherapy-induced PN be conducted. Additionally, cross-sectional nature of this study is another limitation. Although we investigated correlations in addition to cross-sectional comparisons, a future study that investigated longitudinal associations between concern about falling and physical activity outcomes is recommended in order to confirm the results from this study. This study used a validated wearable sensor to extract physical activity parameters of interest without re-evaluating its validity. Thus, there may be some inaccuracy in estimation of parameters of interest. This study examined the impact of concern about falling on measurable physical activities using a validated pendant sensor, which is limited to cumulated posture duration (sitting, standing, walking, and lying) and daily walking performance (e.g., step count, walking bout count). However, other measurable activity patterns not explored in this study (e.g., sleep quality, sedentary behavior, etc.) may better serve as digital biomarkers of concern about falling. In addition, this study did not explore the direct association between identified digital biomarkers of concern about falling and its consequences such as risk of fall. Although this study excluded subjects with potential confounding factors such as Parkinson’s disease, dementia, and active foot ulcer or foot infection, and controlled for some of major confounders such as PN symptoms and age, which might affect daily physical activities, there still may exist effects from other uncontrolled confounders that might affect the patterns of physical activities.

## 5. Conclusions

In conclusion, this study suggests that, among people with PN, daily physical activity patterns measured using a pendant deteriorate with the severity of concern about falling regardless of PN severity. However, only metrics associated with a number of daily walking bouts and step counts reached a statistically significant level in our sample. People with PN and low concern about falling tended to have more activity, but people with PN and high concern about falling tended to have less activity. Furthermore, the duration and amount of being active (i.e., walking bout and total step counts) may predict the level of concern about falling, and thus may be used as eHealth targets and strategies for fall risk assessment among people with PN.

## Figures and Tables

**Table 1:** Participants’ demographic and clinical characteristics.

Measures	All	Low	Moderate	High	*p*-Value *
Sample, N	49	15	12	22	-
Age, years	68.5 ± 7.1	68.4 ± 7.5	67.3 ± 7.7	69.3 ± 6.8	0.666
Women, N (%)	21 (42.9%)	5 (33.3%)	4 (25.0%)	13 (59.1%)	0.106
BMI, kg/m^2^	30.01 ± 5.83	30.18 ± 3.94	26.54 ± 4.08	31.79 ± 6.95	0.039
FES-I, no unit	30.1 ± 13.4	17.1 ± 1.2	23.6 ± 2.7	42.5 ± 10.1	<0.001
Falls, N †	0.7 ± 1.1	0.1 ± 0.3	0.6 ± 1.0	1.1 ± 1.4	0.009
Fallers, N †	17 (34.7%)	1 (6.7%)	4 (33.3%)	12 (54.5%)	0.011
VPT, volts	27.5 ± 11.9	26.2 ± 12.3	26.6 ± 11.4	28.8 ± 12.4	0.796

*Abbreviations:* BMI = body-mass index; FES-I = falls efficacy scale-international; VPT = vibration perception threshold; *Note:* Values represent mean ± standard deviation. * *p*-values denote statistical differences among the group with low concern about falling (FES-I ≤ 19), the group with moderate concern about falling (20 ≤ FES-I ≤ 27), and the group with high concern about falling (FES-I ≥ 28) based on one-way ANOVA (BMI, VPT), Kruskal–Wallis tests (age, FES-I, number of falls), and Chi-square tests (number of women, number of fallers). A *p*-value < 0.05 was considered a significant difference among the two groups. † Falls and fallers represent the number of falls in the past 12 months and the number of people who had a fall incidence in the past 12 months, respectively, based on participants’ self-reports.

**Table 2:** Characteristics of walking behavior.

Measures, N	All	Low	Moderate	High	*p*-Value *
Total walking bouts	440 ± 36	548 ± 79	417 ± 76	378 ± 38	0.119
Total step counts	9377 ± 826	11836 ± 1668	8990 ± 1462	7912 ± 1137	0.123

*Note:* Values represent mean ± standard error. * *p*-values denote statistical differences among the three groups based on one-way ANOVA.
